# UVC-Induced Stress Granules in Mammalian Cells

**DOI:** 10.1371/journal.pone.0112742

**Published:** 2014-11-19

**Authors:** Mohamed Taha Moutaoufik, Rachid El Fatimy, Hassan Nassour, Cristina Gareau, Jérôme Lang, Robert M. Tanguay, Rachid Mazroui, Edouard W. Khandjian

**Affiliations:** 1 Centre de recherche, Institut universitaire en santé mentale de Québec, Département de psychiatrie et de neurosciences, Université Laval, Québec, PQ, Canada; 2 Centre de recherche du CHU de Québec. Département de biologie moléculaire, biochimie médicale et pathologie, Université Laval, Québec, PQ, Canada; 3 Laboratoire de génétique cellulaire et du développement, Département de biologie moléculaire, biochimie médicale et pathologie, Université Laval, Québec, PQ, Canada; German Cancer Research Center, Germany

## Abstract

Stress granules (SGs) are well characterized cytoplasmic RNA bodies that form under various stress conditions. We have observed that exposure of mammalian cells in culture to low doses of UVC induces the formation of discrete cytoplasmic RNA granules that were detected by immunofluorescence staining using antibodies to RNA-binding proteins. UVC-induced cytoplasmic granules are not Processing Bodies (P-bodies) and are *bone fide* SGs as they contain TIA-1, TIA-1/R, Caprin1, FMRP, G3BP1, PABP1, well known markers, and mRNA. Concomitant with the accumulation of the granules in the cytoplasm, cells enter a quiescent state, as they are arrested in G_1_ phase of the cell cycle in order to repair DNA damages induced by UVC irradiation. This blockage persists as long as the granules are present. A tight correlation between their decay and re-entry into S-phase was observed. However the kinetics of their formation, their low number per cell, their absence of fusion into larger granules, their persistence over 48 hours and their slow decay, all differ from classical SGs induced by arsenite or heat treatment. The induction of these SGs does not correlate with major translation inhibition nor with phosphorylation of the α subunit of eukaryotic translation initiation factor 2 (eIF2α). We propose that a restricted subset of mRNAs coding for proteins implicated in cell cycling are removed from the translational apparatus and are sequestered in a repressed form in SGs.

## Introduction

Stress granules (SGs) are transient entities that appear in the cytoplasm when cells encounter certain uncomfortable situations such as thermal shock, viral infection and oxidative stress conditions. Current evidence suggests that SGs are formed as a result of stress-induced translation initiation repression, thereby sequestering mRNAs in an untranslated form [1]–[3]. This is believed to free ribosomes needed for efficient and rapid translation of mRNAs whose products are required to respond to the stress. An other model posits that mRNA are sequestered and protected temporally in granules until the cell recover normal physiological conditions. Both proposals are compatible from a holistic point of view.

In addition to mRNA, SGs contain 40S ribosomal subunits, translation initiation factors such as eIF4G, and RNA-binding proteins (RBPs) that regulate translation [4]. SGs also contain RBPs involved in mRNA reprogramming that could contribute to the reported role of SGs in mRNA protection [5]. Some SGs-associated proteins are also known to be components of processing-bodies (P-bodies) [6]–[8]. However, unlike SGs, P-bodies are present under normal physiological conditions and are believed to serve as mRNA decay sites [3], [4]. Both the number and size of P-bodies increase upon stress-mediated inhibition of translation initiation, suggesting that they also are sites where mRNAs are targeted for translation silencing [7]–[9]. Following stress, PBs and SGs appear to be adjacent, raising the possibility that a trafficking between both entities may exist. This trafficking may be required to coordinate both translation repression and mRNA degradation pathways, in order to ensure an adequate cell response to stress.

It has been reported that most of SGs-inducing stresses inhibit translation initiation through phosphorylation of the translation initiation factor eIF2α at Ser51 [10]. Stress-induced phosphorylation of eIF2α prevents its association with the initiator tRNA, thereby inhibiting translation initiation by stalling initiation complexes in an inactive form. The accumulation of such complexes is believed to result in the formation of SGs. However, this can also occur independently of phosphorylation of eIF2α. For example it was shown that inhibition of translation initiation rates by targeting the activity of the initiation factor eIF4A with either pateamine or hippuristanol is sufficient to induce SGs [11]–[13]. Also, RNA granules resembling SGs were shown to be induced independently of eIF2α phosphorylation following overexpression of specific RBPs such as G3BP1 [14] and FMRP [15] in mammalian cells. More recent studies showed that overexpression of the RBP protein SDC6 induces formation of SG-like RNA granules without eliciting translation repression [16]. These studies suggest that formation of SGs may be uncoupled from inhibition of translation initiation. Consistent with these proposals, it has been reported using the yeast model, that UVC irradiation induces the formation of a new class of RNA granules while no inhibition of translation initiation was observed [17]. On the other hand, although it has been reported that UVC can induce SG-like granules in mammalian cells [18]–[20], the identity of these granules remains elusive.

In the present study, we sought to characterize the cytoplasmic granules induced by UVC in mammalian cells. We present evidence that these radiations trigger the formation of small cytoplasmic RNA granules that contain classical SG components such as TIA-1, TIA-1/R, Caprin1, FMRP, G3BP1, PABP1, known markers, and mRNA. However, their formation and decay occur with different kinetics as compared to classical SGs induced by heat or arsenite treatments. Interestingly, the induction of SGs by UVC does not correlate neither with major translation inhibition nor with eIF2α phosphorylation, despite that their formation coincides with arrest of DNA synthesis and cell proliferation.

## Results

During an unrelated study on cross-linking of Fragile X mental retardation protein (FMRP) to its target RNAs by UVC irradiation [21], we observed that surviving cells irradiated with low doses of UVC, contained FMRP positive cytoplasmic granules as revealed by immunofluorescence staining with antibodies to FMRP. Since the induction of cytoplasmic granules following UVC irradiation is poorly documented, we sought to characterize these cytoplasmic granules in view of their possible relation to SGs.

### UVC induces the formation of small cytoplasmic granules and affects cell proliferation of mammalian cells in a dose-dependent manner

Despite the fact that it has been reported as early as 1999 that irradiation of cells in culture with UVC induces SGs [10], little if any studies have advanced our understanding on how and why these ionizing radiations induce the formation of these cytoplasmic structures. A major handicap is the fact that a wide range of UVC irradiation regimes, from weak to strong [22], can be applied to the cells, most of them resulting in cell death, therefore precluding any further studies on living cells.

**Figure 1:**
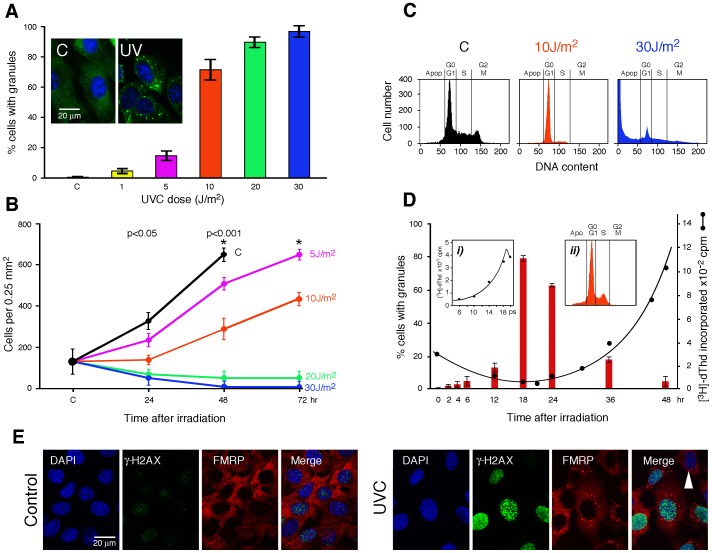
UVC induces the formation of small cytoplasmic granules and affect cell proliferation. **A**) NIH-3T3 cells were treated with 5, 10, 20, and 30 J/m^2^ UV at 254 nm. Eighteen hours post-irradiation, cells were fixed, permeabilized, and processed for immunofluorescence using mAb1C3 against FMRP. Nuclei were counterstained with DAPI. The percentage of cells containing granules was determined from four different experiments each at different irradiation doses. A total of 2000 cells was observed for each dose. Error represents 1 SD calculated from 4 sets of independent experiments. **B**) Proliferation kinetics of NIH-3T3 seeded on coverslips with engraved grids after irradiation at different doses of UVC (5, 10, 20 and 30 J/m^2^). Cells were photographed with a camera on an inverted microscope equipped with phase contrast. The number of cells on each grid was counted from five different experiments. Error represents 1 SD calculated from 5 sets of independent experiments. Stars indicate 100% confluence. **C**) Cell cycle distribution of NIH-3T3 showing a complete G_1_ arrest in cells exposed to 10 J/m^2^ and fragmented DNA (apoptotic: Apo) after a 20 J/m^2^ irradiation. Cells were analyzed 18 h after irradiation. **D**) Correlation between the presence of granules and DNA synthesis in cells exposed to 10 J/m^2^. Quiescent serum-starved NIH-3T3 cells were irradiated and then serum stimulated. At different times after irradiation, cells were fixed, permeabilized, and then processed for immunofluorescence using antibodies against FMRP. The number of cells containing granules was counted from three different experiments containing a total of 3000 cells. Also shown is the rate of incorporation of [^3^H]-Thd into trichloroacetic acid precipitable material after a 30 minutes pulse labeling. Insert i): time course of serum-induced DNA synthesis in synchronized control cells. Insert ii): FACS analysis showing cells re-entering S-phase at 48 hours post-irradiation. **E**) Eighteen hours post-irradiation, NIH-3T3 cells were fixed, permeabilized, and processed for immunofluorescence using antibodies against FMRP (red) and γ-H2AX (green) to determine the formation of FMRP granules and the extend of double-strand breaks induced by UVC irradiation. Nuclei were counterstained with DAPI. The arrowhead in the merged image (right panel) points to a cell that shows very weak γ-H2AX staining and no FMRP granules.

To determine the optimum conditions to study these granules, we followed the fate of FMRP as a marker. This cytoplasmic protein is an RNA binding protein with a diffuse localization in the cytoplasm and can rapidly accumulate in SGs in response to various stresses [23]. UVC irradiation of NIH-3T3 cells at 254 nm relocates FMRP in cytoplasmic foci (Figure 1A). Surprisingly, we were quite disoriented at first since we were not able to detect any cytoplasmic granules at 30, 60 and 90 minutes after treatment, as it is the case for arsenite or heat shock stresses. We therefore prolonged our observations up to 18 hours and detected these structures as an unexpected late event. In cells exposed to 5 J/m^2^ only 20% of the cells contained granules 18 hours after irradiation. Increasing the dose to 10 J/m^2^ resulted in 80% cells containing granules and a maximum of 100% was reached with 30 J/m^2^ (Figure 1A). Similar results were obtained using human HeLa and murine C2C12 cells (data not shown), indicating that formation of these granules is not restricted to murine fibroblasts. Since it is well known that UVC induces cell cycle arrest and cell death [24]–[27] depending on the radiation dose used, we tested the best conditions that would allow us to follow the cell response in living cells. To determine with accuracy the survival rate of the cells exposed to different doses of UVC, NIH-3T3 cells were seeded on coverslips carrying photoeched engraved alphanumeric coded square grids. Through time-lapse imaging under the microscope, we were able to follow initial cell clusters from their original location and thus to determine the number of cells, and their doubling potential.

We observed that UVC irradiation at 20 and 30 J/m^2^ resulted in a significant loss of cells attached to the coverslips. At 48 h post-irradiation, only 5–10% of the cells remained still present as compared to the initial number of cells seeded as control and which in the mean time have doubled their population (Figure 1B). In contrast, the number of cells exposed to a 10 J/m^2^ regime remained slightly constant at 48 hours while later the number of cells present in the same clusters started to increase steadily. Since the number of cells exposed to 10 J/m^2^ did not changed after 24 h, we inferred that they were blocked in the G_1_ phase of the cell cycle, a phenomenon that has been described for a while [24]–[27]. In agreement with these observations, FACS analyses clearly showed that a UVC dose of 10 J/m^2^ caused a complete G_1_ arrest, while fragmented DNA was observed in cells irradiated at 30 J/m^2^ (Figure 1C), in line with the fact that these cells went into an apoptotic process as they left their substrates (see above). Thus, the minimal dose (10 J/m^2^) of UVC that induces formation of granules in a high number of cells correlates with cell cycle arrest.

Intriguing enough, while the number of SGs decreases steadily within 30–60 minutes after releasing of the stress treatment, whether heat or arsenite, granules induced by UVC persist late post-irradiation. To elucidate this unexpected behaviour, we followed the kinetics of appearance and decay of these structures over a period of 48 hours, using FMRP as a marker. For these analyses, NIH-3T3 cells were synchronized in G_1_ phase after serum starvation. Quiescent cultures were irradiated with 10 J/m^2^ UVC and immediately replenished with fresh medium containing 10% serum for mitotic stimulation. Time course analyses showed a gradual accumulation of granules starting around 2 hours post-irradiation (2–3% of positive cells), increasing steadily to reach their maximum around 18 hours, at a time when usually NIH-3T3 cells have normally doubled their DNA before mitosis (Figure 1D, insert i). Then after the number of granules decayed steadily (Figure 1D). To determine a possible correlation between the delay in cell proliferation, as discussed above, and the presence of the cytoplasmic granules, the kinetics of DNA synthesis was measured by incorporation of [^3^H]-dThd into acid-insoluble material. In irradiated cells, a drop in [^3^H] incorporation was observed as compared to the control (Figure 1D, insert i), and a drastic delay in S-phase was observed as incorporation of the radio-active DNA precursor was shifted by approximately 24 h. Confirming these results, FACS analyses showed that indeed an increase of S-phase occurred at 48 hours as compared to the results presented in Figure 1C. Concomitant to the recovery of DNA synthesis, a clear decrease of granules was observed (Figure 1D), thereby establishing a correlation between granules disassembly and re-entry into the cell cycle. Double immunostaining with anti-FMRP and anti-γ-H2AX, a key factor in the repair process of damage DNA [28]–[30], clearly showed a tight correlation between DNA repair and the presence of granules (Figure 1E).

Altogether, these results show that UVC induces formation of reversible granules in mammalian cells, and that appearance of these foci coincides with cell cycle arrest. We therefore used 10 J/m^2^ of UVC, as this was apparently a non-lethal dose that induced granules in a high percentage of cells, a requirement for biochemical studies.

### Characterization of UVC-induced SGs in mammalian cells

**Figure 2:**
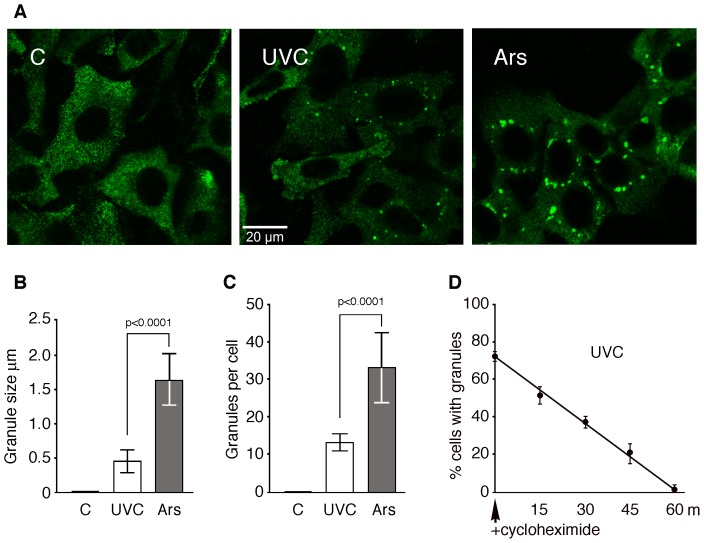
Physical comparisons between UVC-induced granules and *bone fide* SGs. **A**) Control, UVC-irradiated and arsenite treated NIH-3T3 cells were processed for immunofluorescence to detect FMRP. **B**) Size of the granules induced by UVC was determined 18 hours post-irradiation (n = 2000) and those induced by arsenite after 60 minutes post-treatment (n = 2000; P value <0.0001). **C**) Number of granules per cell was determined 18 hours post-irradiation and after 60 minutes arsenite treatment. **D**) Addition of cycloheximide (50 µg/ml) induced UVC-granules to vanish in less than 60 minutes.

Using FMRP as a marker, we determined by immunofluorescence staining that the UVC –induced granules are smaller in size (≈0.5 µm) than the classical SGs such as those triggered by arsenite, which usually range between 1.5 and 2 µm (Figure 2A and B). The average number of granules per cell was also significantly lower in the case of UVC irradiation as compared to the number of SGs induced by arsenite (Figure 2A and C). However, addition of cycloheximide resulted in disappearance of the granules within 60 min (Figure 2D) as it is the case for SGs [23], [31]. Since arsenite also induces formation of P-bodies [6]–[8], which are similar in size to UVC-induced granules, we therefore tested if the latter would correspond to P-bodies.

**Figure 3:**
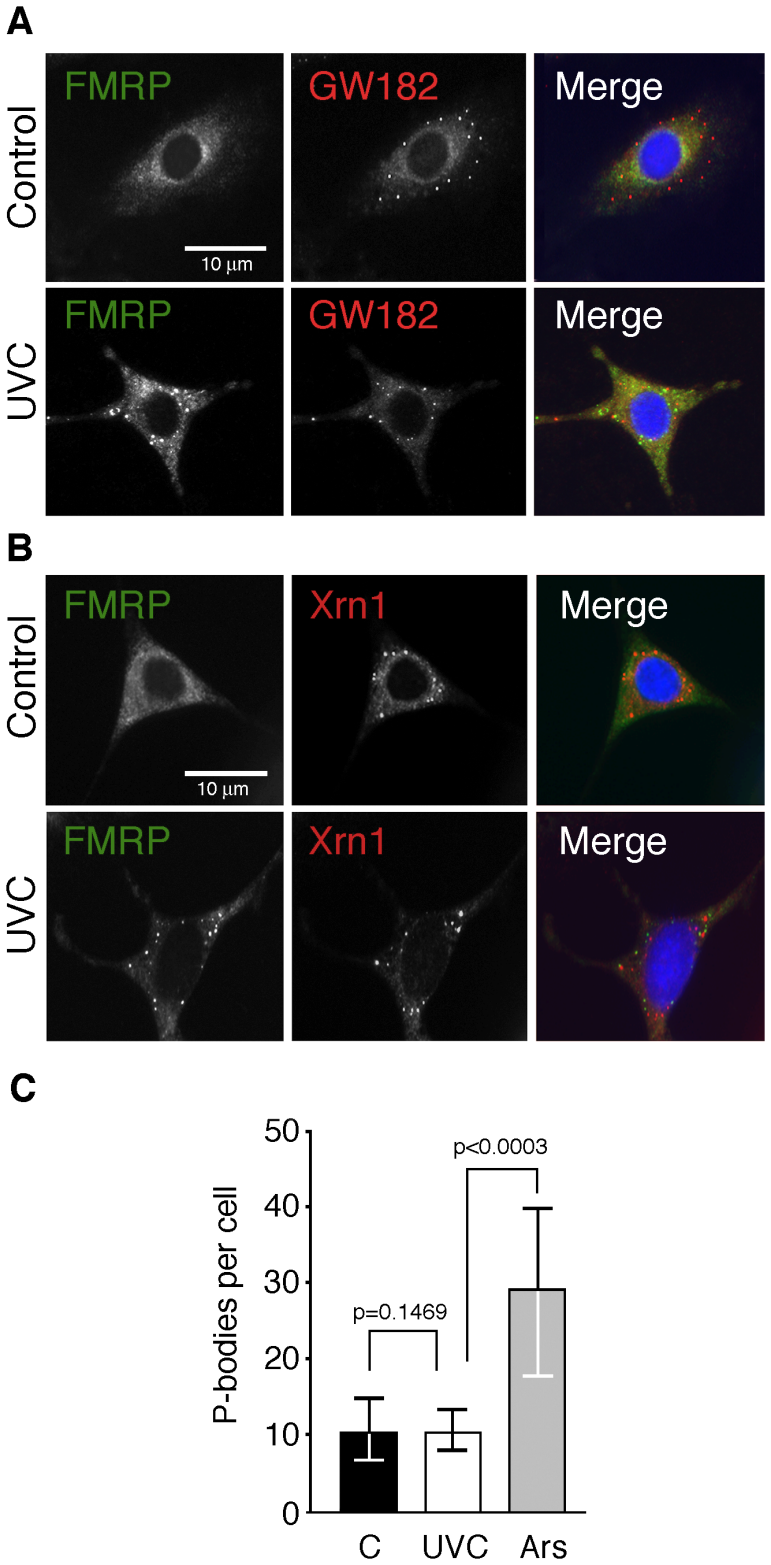
UVC-induced granules do not correspond to P-bodies. **A**) Irradiated (10 J/m2) NIH-3T3 cells were fixed, permeabilized and processed for immunofluorescence using antibody against FMRP (green), and the P-bodies markers GW182 (red). **B**) Double-staining for FMRP (green) and Xrn1 (red). Nuclei were counter-stained with DAPI. **C**) Number of P-bodies per cell remains constant after UVC irradiation (p not significant), while it almost tripled after arsenite treatment (p<0.0003).

Cells were double stained with FMRP and GW182 or Xrn1, two P-bodies specific markers [8], [32]. In control cells, the results clearly showed that P-bodies could be detected as cytoplasmic foci while FMRP was evenly distributed in the cytoplasm (Figure 3A). On the other hand, in UVC irradiated cells FMRP and GW182 were present in cytoplasmic granules and the merge images clearly showed that these granules were independent entities. The same conclusions were obtained after double staining with FMRP and Xrn1 (Figure 3B). While exposure to arsenite increases the number of these granules, we did not detected significant difference in the number of P-bodies before or after UVC irradiation (see Figure 3C). Collectively, these results clearly indicate that the UVC-induced granules do not correspond to P-bodies.

**Figure 4:**
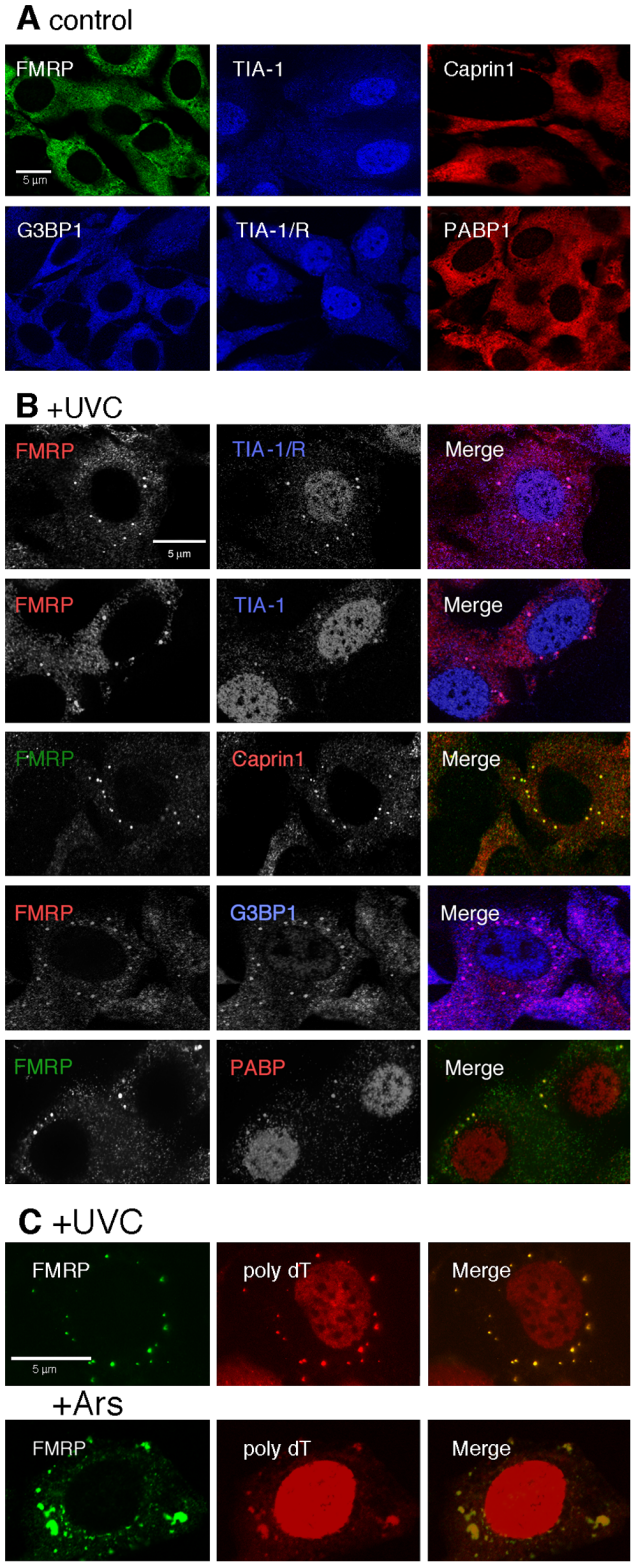
UVC-induced granules correspond to SGs. **A**) Shown are the locations of the SGs protein markers in NIH-3T3 cells grown under normal conditions. **B**) Cultures were UVC irradiated (10 J/m2) and cells were fixed, permeabilized and processed for immunofluorescence to detect FMRP (red or green), TIA-1/R and TIA-1 (blue), Caprin1 (red), G3BP1 (blue) and PABP1 (red). Note the translocation of G3BP1 and PABP1 into nuclei. Because some of the SGs signals were not easily detected over a high cytoplasmic background, especially in the blue range, shown here are the black and white original images for all single channel pictures, while the merge images are shown in colors. **C**) Cultures were treated with a buffer containing 1% NP40 to release most of the cytoplasmic fraction and processed for *in situ* hybridization with poly-dT followed by staining for FMRP using mAb1C3.

We then tested the second possibility whether UVC-induced granules correspond to SGs. We therefore searched for the presence of canonical SGs complements such as TIA-1 and TIA-1/R [10], Caprin1 [33] that interacts physically with FMRP [34], G3BP1 [35] and PABP1 [10]. As expected, in control cells, TIA-1 and TIA-1/R were predominantly nuclear, while FMRP, Caprin1, G3BP1 and PABP1 were evenly distributed in the cytoplasms (Figure 4A). Eighteen hours post-irradiation, all SGs markers were found in cytoplasmic granules. Because of their small size, these UVC-induced granules were difficult to detect in some cases especially in the blue channel. Therefore to enhance the contrast, black and white images are presented for single channel pictures (Figure 4B). Unexpectedly, in approximately 20% of cells containing granules, G3BP1 was also detected in nuclei, as was also the case for PABP1. Since the ultimate function of the SGs is to entrap and protect temporarily mRNA, we tested whether this is also the case for UVC-induced granules. However, we were not able to visualize by FISH any foci due to strong cytoplasmic stainings after poly-dT hybridization. To reduce the background staining, we applied a method to obtain a cell ghost monolayer [36],[37]. As classical SGs are known to remain associated with the cytoskeleton framework [23] it was possible to remove, through washes, the majority of the cytoplasmic components. UVC irradiated as well as arsenite treated NIH-3T3 cells grown on coverslips were incubated *in situ* on ice for 10 min in an extraction buffer containing 1% NP-40. After slowly removing the soluble extracts, the remaining materials were fixed and processed for FISH analyses followed by immunofluoresence staining to detect FMRP. After such a treatment, FISH analyses clearly showed that granules induced by UVC irradiation detected by FMRP, also contain poly(A)^+^ mRNAs (Figure 4C). Altogether these results identified UVC-induced granules *as bone fide* SGs.

### Formation of UVC-induced SGs occurs independently of eIF2α phosphorylation and does not require major inhibition of translation initiation

**Figure 5:**
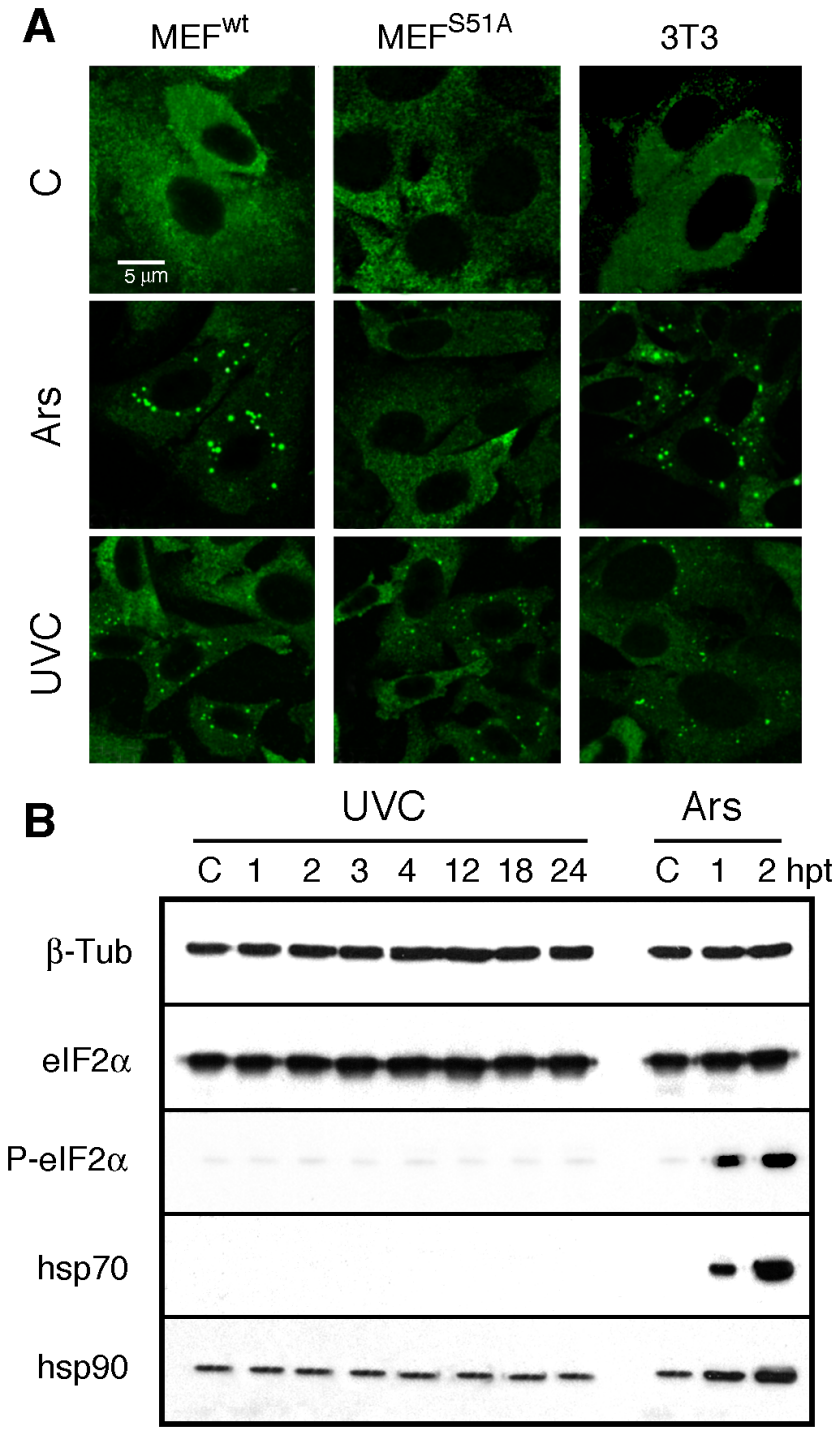
UVC-induced SGs are formed independently of eIF2α phosphorylation. **A**) SGs are present in UVC-irradiated NIH-3T3, MEF^wt^ and MEF^S51A^ while they are not formed in MEF^S51A^ cells treated with arsenite. Cells were fixed, permeabilized, and then processed for immunofluorescence using mAb1C3 against FMRP. **B**) Immunoblot analyses of MEF^wt^ showing that eIF2α is not phosphorylated and that HSP90 and 70 are not induced after UVC irradiation, while all three proteins react after arsenite treatment. Tubulin β was used as a control for even loading of samples. C: control; hpt: hours post-treatment.

While phosphorylation of eIF2α at serine 51 (S51) appears to be the major mechanism by which SGs are formed by various stresses [10], exposure to low levels of UVC such as those used in the present study did not induce its phosphorylation [18], [38]; these observations are in line with results showing that a minimum of 50–60 J/m^2^ was required to induce eIF2α phosphorylation [39]. We therefore wished to illustrate the uncoupling of phosphorylation of eIF2α from UVC-induced SGs formation using MEF cells derived from homozygous eIF2α knockin mouse embryo expressing a non-phosphorylatable eIF2α mutant (MEF^S51A^) [40]. These cells are unable to form SGs upon arsenite treatment. Using these mutant cells, we observed that UVC-SGs are induced as efficiently as in MEF^wt^ as well as in NIH-3T3 cells (Figure 5A). Immunoblot analyses performed on extracts from MEF^wt^ exposed to UVC or treated with arsenite clearly revealed that formation of SGs does not require the activation of the eIF2α phosphorylation response. Consistent with these results, we also observed that UVC treatment of MEF^wt^ cells does not induce the expression of stress heat shock proteins (Figure 5B), further indicating that formation of these SGs is not caused by a general stress response.

**Figure 6:**
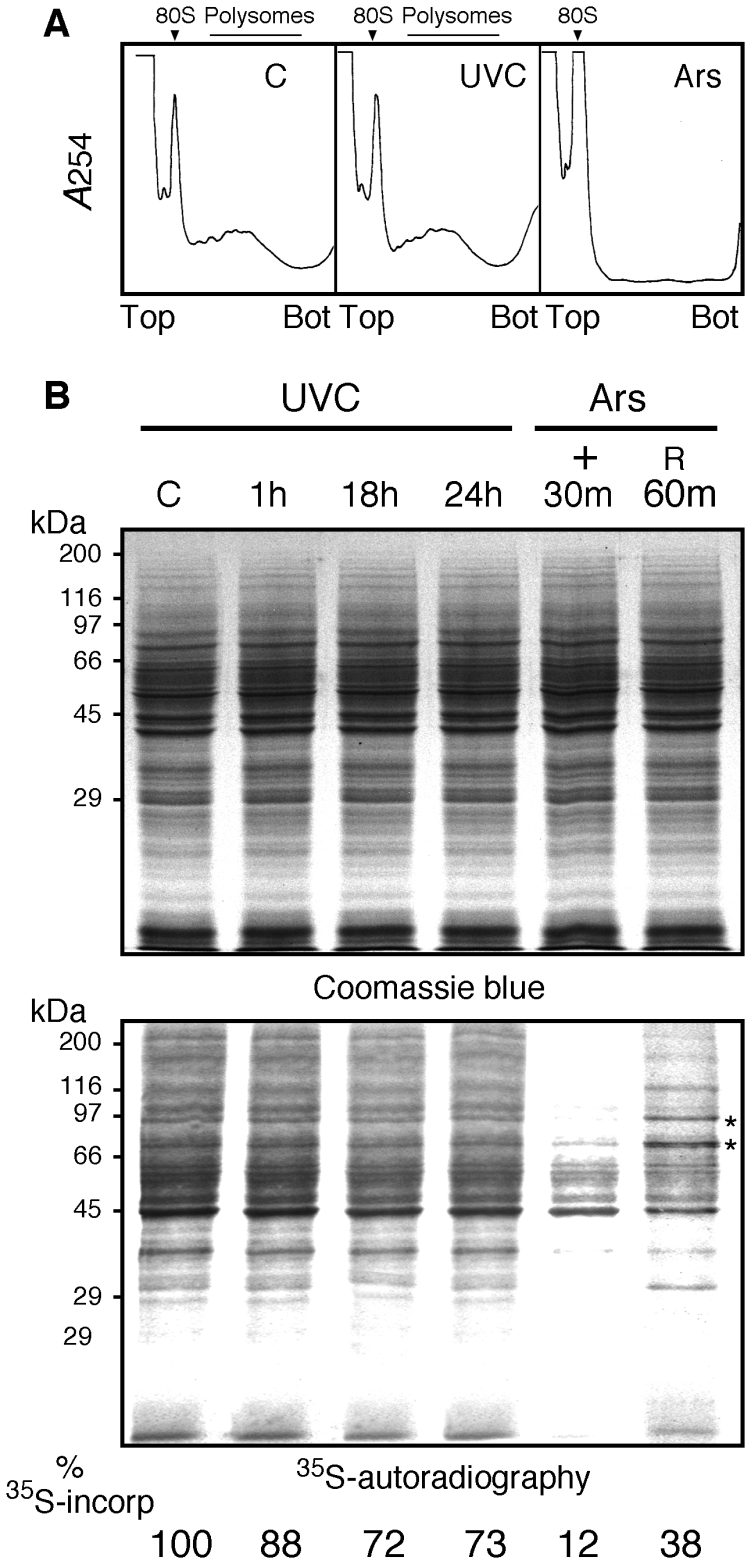
Effects of UVC on translation. **A**) Polyribosome profiles of control NIH-3T3 and cultures after UVC irradiation with 10 J/m^2^ or exposure to sodium arsenite. Cells were extracted 18h after UV irradiation or after 30 minutes of arsenite treatment and the cytoplasmic fractions analyzed by sedimentation in sucrose density gradients. The sedimentation position of the 80S ribosomes is indicated by an arrow. **B**) Newly synthesized proteins detected by incorporation of ^35^S-methionin/cystein in cells exposed to sodium arsenite or irradiated with UVC. Cells were labelled 30 minutes in the presence of 50 µCi/ml [^35^S]-methionine/cystein in methionine-free DMEM. Total cell extracts (∼40 µg protein) were analyzed on a 11% SDS-PAGE. Upper panel: Coomassie blue staining. Lower panel: the same gel was dried and exposed to an X-ray film. Stars indicate the positions of hsp90 and hsp70.

Since most stress-inducing SGs are known to inhibit translation initiation, we tested if formation of SGs by UVC correlates with translation repression. First, we performed polyribosome analyses 18 h after UVC irradiation, when the number of SGs is at its maximum. Cytoplasmic extracts from control, irradiated and arsenite treated NIH-3T3 cells were fractionated on sucrose gradient and absorbance at 254 nm was continuously monitored (Figure 6A). While arsenite treatment completely disrupted polyribosomes indicating a general inhibition of translation, no noticeable changes in the polyribosomes profiles were observed after UVC irradiation. Since these analyses do not reflect subtle changes in the translation status, a ^35^S incorporation assay was used to quantify the rate of protein synthesis after irradiation. NIH-3T3 cells were metabolically labelled with [^35^S]-Met/Cyst at different times post-irradiation. As a control for protein synthesis inhibition, cells were also labelled for 30 minutes after a 30 minutes treatment with arsenite as well as after 60 minutes of recovery in culture medium without the drug. SDS-PAGE analyses clearly showed that while protein synthesis in the presence of arsenite was reduced by 88%, only a 27% decrease was observed for UVC-irradiated cells after 24 hours (Figure 6B). This decrease was noticeable as early as 1 hour after irradiation (Figure 6B and Figure S1). Note that the *de novo* labelled protein detected at 70 and 90 kDa corresponding to the major HSPs of cells resuming protein synthesis after the release of arsenite blockage are not present in extracts of UVC-irradiated cells (Figure 6B). Collectively, these observations support the notion that formation of SGs by UVC occurs without major inhibition of general translation.

## Discussion

Treatment of mammalian cells with UVC induces the formation of granules that have been prematurely considered to correspond to SG, however with no formal proofs but the presence of one single marker TIA-1 or TIA-1/R [18], [19]. It has been reported recently that a novel class of mRNA-containing cytoplasmic granules are produced in yeast in response to UV-irradiation [17]. These granules are neither P-bodies nor SGs and the authors proposed that these granules correspond to foci where poly(A)+mRNAs damaged by UVC irradiation are stocked although, the widely accepted concept is that damaged RNA is degraded rather than repaired.

In the present study, we observed that UVC-induced SGs are smaller and less numerous per cell than the bona fide SGs induced by arsenite or heat treatment, and surprisingly that they are not formed as an early response to the stress since they accumulate late after the treatment, in contrast to other reports that show a maximum peak between 1–4 hours post-irradiation [18], [19]. These SGs lack P-body components but contain canonical SG markers including mRNA. Unexpectedly, while all SG markers tested here co-localize in granules, PABP1 and G3BP1 were also detected in approximately 20% of the nuclei. The translocation of PABP1 to the nucleus has been previously observed after UVC irradiation of HeLa and NIH-3T3 cells and it has been proposed that this phenomenon might be part of the cellular strategy to control translation, leaving unanswered the question why certain cellular stresses such as UVC irradiation [20] relocalize PABP1 to the nucleus rather only to SGs. Concerning the presence of G3BP1 in the nucleus, it is worth mentioning that this location has been observed in rubella virus infection [41] and after UVC irradiation of HeLa cells [42] but unfortunately this phenomenon has not been further explored. Of high interest is that in cells exposed to methyl-N9-nitro-N9-nitrosoguanidine (MNNG), an alkylating agent that induces DNA damages, G3BP1 is found in SGs as well as in nucleus [43]. These findings as well as our's, are in contradiction with the current accepted notion that ionizing radiations as well as genotoxic drugs (Type 2 stress) do not trigger the formation of SGs [44]. Our results showing that SGs co-exist with γ-H2AX nuclear foci, markers of DNA damages [28]–[30] are in contradiction with the results presented by Pothof et al. [19] who reported the absence of SGs in cells that showed bright γ-H2AX staining. Also, while these authors reported that only 15–20% of the HeLa cells formed SGs in asynchronous growing cells, we constantly observed in HeLa, NIH-3T3, MEF^wt^, MEF^S51A^, and C2C12 cells a 60–80% cells carrying SGs at 18 hours post-irradiation.

Because of the small size of UVC-induced SGs, we suggest that these structures contribute to regulate the expression of only a restricted subset of mRNAs. This assumption is based on the fact that relatively few SGs are formed per cell upon UVC exposure making unlikely the possibility that they play a general role in mRNA regulation. Along the same line, the “mild” UV conditions used here did not induce the expression of the classical stress heat shock proteins and no total inhibition of general translation initiation was observed. It is worth emphasizing that under the conditions used here, in NIH-3T3 and MEF^wt^ cells irradiated at 10 J/m^2^, only a decrease of 27% in newly protein synthesis was observed at 24 hours after irradiation, matching with previous data using MCF-cells (5% and 29% at 5 and 15 J/m2 respectively) [38]. Also, Deng et al. [39] have observed that a dose of at least 80 J/m2 was required to detect a 20% decrease of ^35^S-methionine incorporation into newly synthesized proteins. Our results are consistent with the observation that UV-radiation elicits different dose-dependant cellular response. For instance a 10 J/m2 UVC irradiation results in a transient decrease of approximately 24 hours in DNA-replication, while a 50 J/m2 dose induces a permanent cessation of DNA replication [26].

The cellular response to UVC irradiation is an extremely complex process and involves several steps to identify and to restore the damaged DNA. These processes include genome integrity surveillance, DNA damage recognition and DNA repair, cell signalling and cell cycle arrest giving the time to the cell to repair the DNA before resuming proliferation [24]–[27]. Protein synthesis is therefore required to perform all these complex steps and it is unconceivable that the presence of SGs corresponds to a general translation arrest. Our results clearly demonstrate that the presence of SGs coincides with S-phase arrest. Cells remain in G_1_-phase as long as granules are present, or perhaps vice versa. A correlation was observed between decay of SGs and re-entry into S-phase. Based on these observations we propose that the appearance of SGs does not coincide with global translation arrest, but instead that unneeded mRNAs coding for proteins implicated in cell cycle progression are withdrawn from the translation apparatus in order to be stored as silent entities in granules. Once DNA damages are repaired, these silent mRNAs are released from the granules and are moved back to the translation machinery.

In conclusion, we propose that UVC induces the recruitment of a restricted subset of mRNAs and associated proteins from active polyribosomes to be stored in a repressed form within SGs. Since their formation correlates with cell cycle arrest, it is tempting to speculate that these SGs may sequester mRNAs directing the synthesis of cell cycle regulator proteins, thereby preventing their expression and thus cell proliferation. Clearly, identification of SGs-associated mRNAs will help to uncover the role of these entities in the control of cell proliferation upon UVC exposure. Although descriptive, we believe that the present study might open new avenues in understanding the cellular response to UVC irradiation.

## Materials and Methods

### Cell Lines and Cultures

NIH-3T3 cell line was purchased from ATCC, wild type mouse embryonic fibroblasts (MEFs) and eIF2α^S51A/S51A^ MEF mutants obtained from Randal J. Kaufman have been described previously [40]. Cells were cultured in DMEM supplemented with 10% fetal bovine serum (FBS), penicillin, and streptomycin. All chemicals were from Sigma-Aldrich. To synchronize cells in G_1_ phase, NIH-3T3 cultures at 50% confluence were serum starved in medium containing 0.2% FBS, and the cells kept in the same medium for 72 h.

### UV Irradiation, Cell counting and Cell cycle analyses

UVC was generated from a UV light source (Philips T UV 15W/G15 T8). UV flux was measured using a UVX digital radiometer (UVP Inc.) equipped with a specific probe for UVC. The culture medium was removed before irradiation and fresh medium added after UV exposure. For cell counting and proliferation studies, equal number of NIH-3T3 cells was seeded on coverslips with engraved marks (Electron Science Microscopy, #72264-18), irradiated with UVC at different doses and incubated at 37°C. At different time after irradiation, cells in selected chosen squares were followed and photographed with a Sony (DSC-S85) camera mounted on an inverted microscope with phase contrast (Zeiss Axiovert-25) using a 4X objective. Acquired images were transferred to Adobe PhotoShop program, printed and the number of cells present in the same identified squares counted.

At the indicated time after UVC irradiation, cells were trypsinized, fixed in 70% ethanol, resuspended in PBS containing 1 mg/ml propidium iodide and 0.1 mg/ml RNase A. Cell cycle distribution was determined by FACS analyses (BD FACS Arial flow cytometer). For DNA synthesis measurements, cells grown on coverslips were labelled for 30 min with 2.5 µCi [^3^H]-dThd (Perkin Elmer). At the indicated time, coverslips were removed from the wells and transferred to 35 mm petri dishes containing 5% trichloro-acetic acid (TCA) and incubated for 15 minutes on ice. After washing three times with cold 5% TCA, radioactivity was measured on duplicate coverslips in scintillation fluid.

### Antibodies

FMRP was detected using hybridoma supernatants from mouse mAb1C3 or with chicken IgY#C10 [37]; β-tubulin with mAbE7 (obtained from the Developmental Studies Hybridoma Bank, University of Iowa). Human serum 18033 reacting with GW182 [32] was obtained from Marvin J. Fritzler (University of Calgary, Canada). Anti-Hsp70 (SPA-818) and anti-Hsp90 (SPA-830) were from Stressgen, and anti-Xrn1 (ab70259) from Abcam. Phospho-specific anti-eIF2α (9721), anti-eIF2 (9722) and anti-PABP1 (4992) were from Cell Signaling Technology. Anti-G3BP-1 (sc-81940), TIA-1 (sc-1751), TIA-1/R (sc-1749) from Santa Cruz Biotech. Anti-Caprin1 (15112-1-AP) from Proteintech Group, and anti-phospho-Histone H2AX (Ser139) (05-636) from Millipore.

### Immunofluorescence analyses

Cells were fixed in 4% paraformaldehyde in PBS for 10 min at room temperature, permeabilized with methanol at −20°C for 5 min, washed twice with PBS and incubated with primary antibodies diluted in 0.1% Tween-20/PBS (PBST) containing 5% BSA, for 1 h at room temperature. After washing with PBST, cells were incubated with IgG (H+L) secondary antibodies. Secondary antibodies were: donkey anti-mouse Alexa 488 and 405, donkey anti-goat Alexa 405, donkey anti-rabbit Alexa 546, and goat anti-chicken Alexa 546 (Invitrogen) for 45 min. Coverslips were mounted in Prolong Gold Antifade mounting media (Invitrogen). Samples were visualized using the LSM 700 confocal laser scanning microscope (Zeiss), equipped with a ZEN 2009 software for image acquisition and analyses. Images were acquired using the following settings: 63X oil objective (zoom 1.0), 0.06 mm for pixel size, and 1.00 airy units as pinhole.

### Polyribosomes analyses

NIH-3T3 cells were grown in 100-mm tissue culture dishes to 80% confluence, harvested, and resuspended in 1 ml of a buffer containing 20 mM Tris, pH 7.5, 150 mM NaCl, 1.25 mM MgCl_2_, 5 U/ml RNAsine (GE Healthcare), EDTA-free protease inhibitor complete cocktail (Roche) and 1 mM dithiothreitol and 1% Nonidet P-40 [45]. After lysis for 15 minutes on ice, the extracts were clarified by centrifugation at 12,000×*g* for 20 min at 4°C, loaded on 15–45% (w/v) linear sucrose gradient and further analyzed as described [45].

### SDS-PAGE and Immunoblot analyses

Total cell extracts were analyzed on a 11% SDS-PAGE. Immunoblot analyses were performed as described [34]. To study *de novo* protein synthesis, cells in a six-well plate were labelled for 30 minutes with 50 µCi [^35^S]-methionine/cystein (Easy Tag Express Protein Labelling mix, Perkin Elmer Life) in 1 ml of methionine-free DMEM (Sigma-Aldrich) supplemented with 10% FBS. Cell extracts were analyzed by SDS-PAGE, and the proteins revealed with Coomassie Brilliant Blue R-250 (Sigma). Gels were then dried and exposed to Fuji X-ray films. Autoradiograms were scanned and analyzed using the ImageJ64 program, and the percentage of [^35^S] signals for each lane were normalized to the corresponding Coomassie stain intensities.

### Ghost monolayers and FISH analyses

NIH-3T3 cells grown on coverslips in 35 mm diameter petri dishes were rinsed twice with cold PBS and lyzed *in situ* in the presence of a buffer containing 20 mM Tris-HCl, pH 7.4, 150 mM NaCl, 1.25 mM MgCl_2_, 1% NP40, supplemented with Protease Inhibitor Cocktail. The petri dish containing the coverslip was gently swirled at 4°C for 10 minutes on an orbital shaker at low speed, and the supernatant was discarded [36], [37]. After washing twice with PBS, the remaining material attached to the coverslips was fixed with 4% paraformaldehyde in PBS for 5 minutes at room temperature, washed twice with PBS and processed for FISH experiments.

*In situ* hybridization to detect poly(A)+ RNA was performed essentially as described [15]. Hybridization was performed in a humid chamber at 42°C, using a custom made 5′-tagged Alexa Fluor 594-oligo [dT]_25_ (Invitrogen, Burlington, ON, Canada) for 16 h. Samples were washed three times with 2×SSC and incubated with Alexa 594-labeled streptavidin (Molecular Probes) in 4×SSC containing 0.1% Triton X-100 for 45 min at room temperature, were washed in 4×SSC, and then reacted with anti–FMRP mAb1C3 in 2×SSC containing 0.1% Triton X-100. After 60 minutes incubation, samples were again washed in 4×SSC and subjected to a final incubation with goat anti–mouse IgG Alexa 488.

## Supporting Information

Figure S1 ***De novo*** **protein synthesis at early time after UVC-irradiation (10 J/m^2^) of NIH-3T3 cells.** For details see Figure 6 in the main text. hpt: hours post-treatment.
(TIF)
