# Supplementary figures and images for: UVC-Induced Stress Granules in Mammalian Cells
Source: PLoS One. 2014 Nov 19;9(11):e112742. doi: 10.1371/journal.pone.0112742 (PMC4237350; doi:10.1371/journal.pone.0112742)

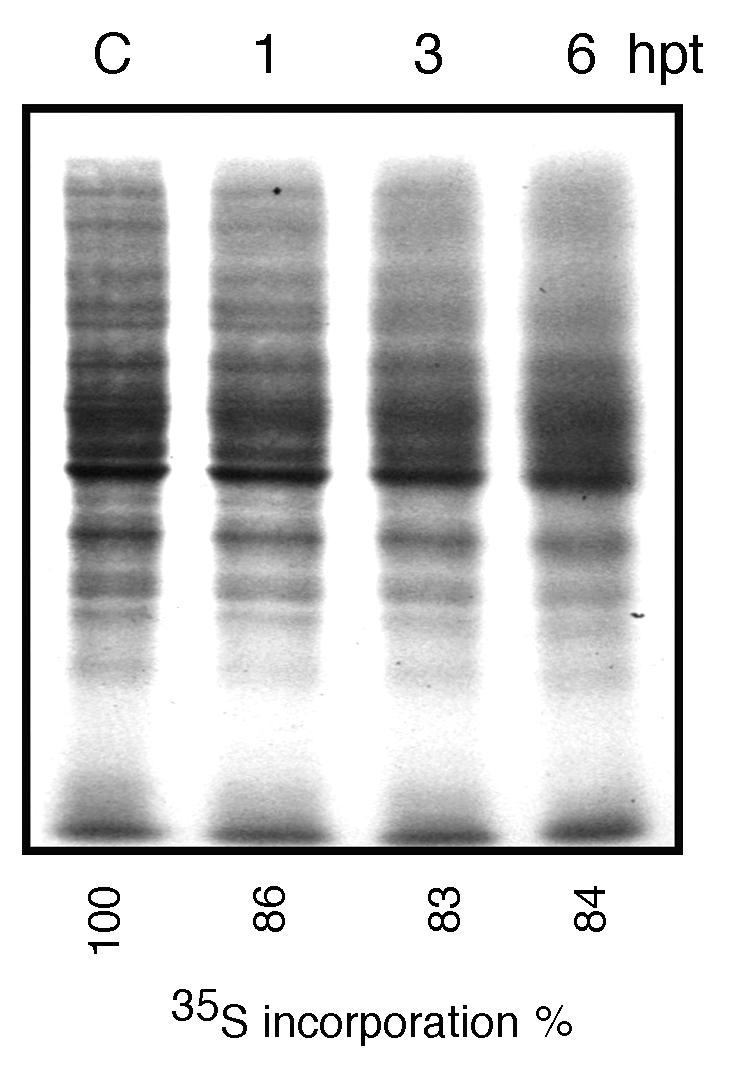

Supplement: Figure S1 — De novo protein synthesis at early time after UVC-irradiation (10 J/m2) of NIH-3T3 cells. For details see Figure 6 in the main text. hpt: hours post-treatment. (TIF) [file pone.0112742.s001.tif]
